# Reaping the benefits of liquid handlers for high-throughput gene expression profiling in a marine model invertebrate

**DOI:** 10.1186/s12896-024-00831-y

**Published:** 2024-01-19

**Authors:** Giovanni Annona, Assunta Liberti, Carla Pollastro, Antonietta Spagnuolo, Paolo Sordino, Pasquale De Luca

**Affiliations:** 1 Research Infrastructures for Marine Biological Resources (RIMAR), Stazione Zoologica Anton Dohrn, Naples, Italy; 2 Biology and Evolution of Marine Organisms (BEOM), Stazione Zoologica Anton Dohrn, Naples, Italy; 3 Biology and Evolution of Marine Organisms (BEOM), Stazione Zoologica Anton Dohrn, Sicily Marine Centre, Messina, Italy; 4 Present Address: TIGEM - Telethon Institute of Genetics and Medicine, 80078 Naples, Italy

**Keywords:** Laboratory automation, Large-scale screening, Automated protocols, *Ciona robusta*, Marine organisms, Immune system, Molecular biology protocols

## Abstract

**Background:**

Modern high-throughput technologies enable the processing of a large number of samples simultaneously, while also providing rapid and accurate procedures. In recent years, automated liquid handling workstations have emerged as an established technology for reproducible sample preparation. They offer flexibility, making them suitable for an expanding range of applications. Commonly, such approaches are well-developed for experimental procedures primarily designed for cell-line processing and xenobiotics testing. Conversely, little attention is focused on the application of automated liquid handlers in the analysis of whole organisms, which often involves time-consuming laboratory procedures.

**Results:**

Here, we present a fully automated workflow for all steps, from RNA extraction to real-time PCR processing, for gene expression quantification in the ascidian marine model *Ciona robusta*. For procedure validation, we compared the results obtained with the liquid handler with those of the classical manual procedure. The outcome revealed comparable results, demonstrating a remarkable time saving particularly in the initial steps of sample processing.

**Conclusions:**

This work expands the possible application fields of this technology to whole-body organisms, mitigating issues that can arise from manual procedures. By minimizing errors, avoiding cross-contamination, decreasing hands-on time and streamlining the procedure, it could be employed for large-scale screening investigations.

**Supplementary Information:**

The online version contains supplementary material available at 10.1186/s12896-024-00831-y.

## Background

In the biotechnological field, the availability of diverse experimental organisms and the presence of fast and reproducible protocols are crucial elements for various research areas, including preclinical screening and ecotoxicological investigations. The advancement of experimental approaches necessitates the development of technologies that optimize execution times and minimize errors. High-throughput platforms have revolutionized the accomplishment of multiple laboratory protocols in a fully-automated manner, ranging from common molecular biology methods to high-throughput screening of bioactive molecules [1, 2]. While automated approaches are well-developed for individual procedures, particularly in cell-line processing for xenobiotics testing [3, 4], less attention has been devoted to sequential experimental procedures and analyses involving whole living organisms. However, the use of model systems remains fundamental for a full comprehension of complex biological processes.

Marine organisms represent an invaluable resource, serving as a rich source of bioactive molecules [5, 6] and as experimental models [7]. Among them, the tunicate *Ciona robusta* (referred to as *Ciona* hereafter) holds great significance in the study of the evolutionary history of chordates, as it belongs to the subphylum phylogenetically closer to Vertebrata [8, 9]. With the availability of in vitro fertilization, embryo manipulation techniques, a fully sequenced genome [10], and the ability to perform gene silencing and genome editing [11, 12], *Ciona* has established itself as a successful experimental system in various research fields, including developmental biology and comparative immunology [13–16]. Concerning the latter, several studies have focused on identifying key components of the *Ciona* innate immune system [16, 17] and the gut mucosal environment. This has also allowed the development of *Ciona* as an experimental model for investigating the role of the immune system in establishing and maintaining gut homeostasis [18–20, 22].

The studies on this experimental organism have paved the way for further development and utilization of *Ciona* as a screening organism for a wide range of xenobiotics and immunomodulators [21–23], investigating the gene modulation within the innate immune system [24]. Recently, it has been demonstrated that automated approaches can be applied to *Ciona* larvae [25, 26]; however, high-throughput conditions have not been achieved yet. In this work, we have set up and tested a fully automated procedure by employing *Ciona* juveniles at stage 4 of metamorphosis when, opening the siphons, they start the interactions with the external environment. This procedure utilizes automated liquid handling systems to execute a comprehensive workflow for sample manipulation, including RNA extraction and purification, RNA normalization, cDNA synthesis, and reverse transcription-quantitative PCR (RT-qPCR) plate assembly.

To validate the effectiveness of the automated procedure, we conducted a step-by-step comparison of automated and manual results. Our experimental setup focused on investigating the gene modulation in the innate immune system following exposure to various microbial stimuli. Specifically, the *Ciona* juveniles were treated with diacyl-lipoprotein Pam2CSK4 and zymosan (components of bacterial and fungal cell walls, respectively), classified as pathogen-associated molecular patterns, known to activate innate immune pathways in both mammals [27, 28] and invertebrates [29].

The achieved results demonstrate the reliability and utility of employing a fully automated workflow for gene expression analysis. They also highlight the strengths and weaknesses of this approach. Consequently, this work showcases the potential of using a whole invertebrate marine organism for large-scale screening of xenobiotics that affect the regulation of immune response genes, as well as other cell signaling pathways or physiological processes.

## Results and discussion

**Fig. 1:**
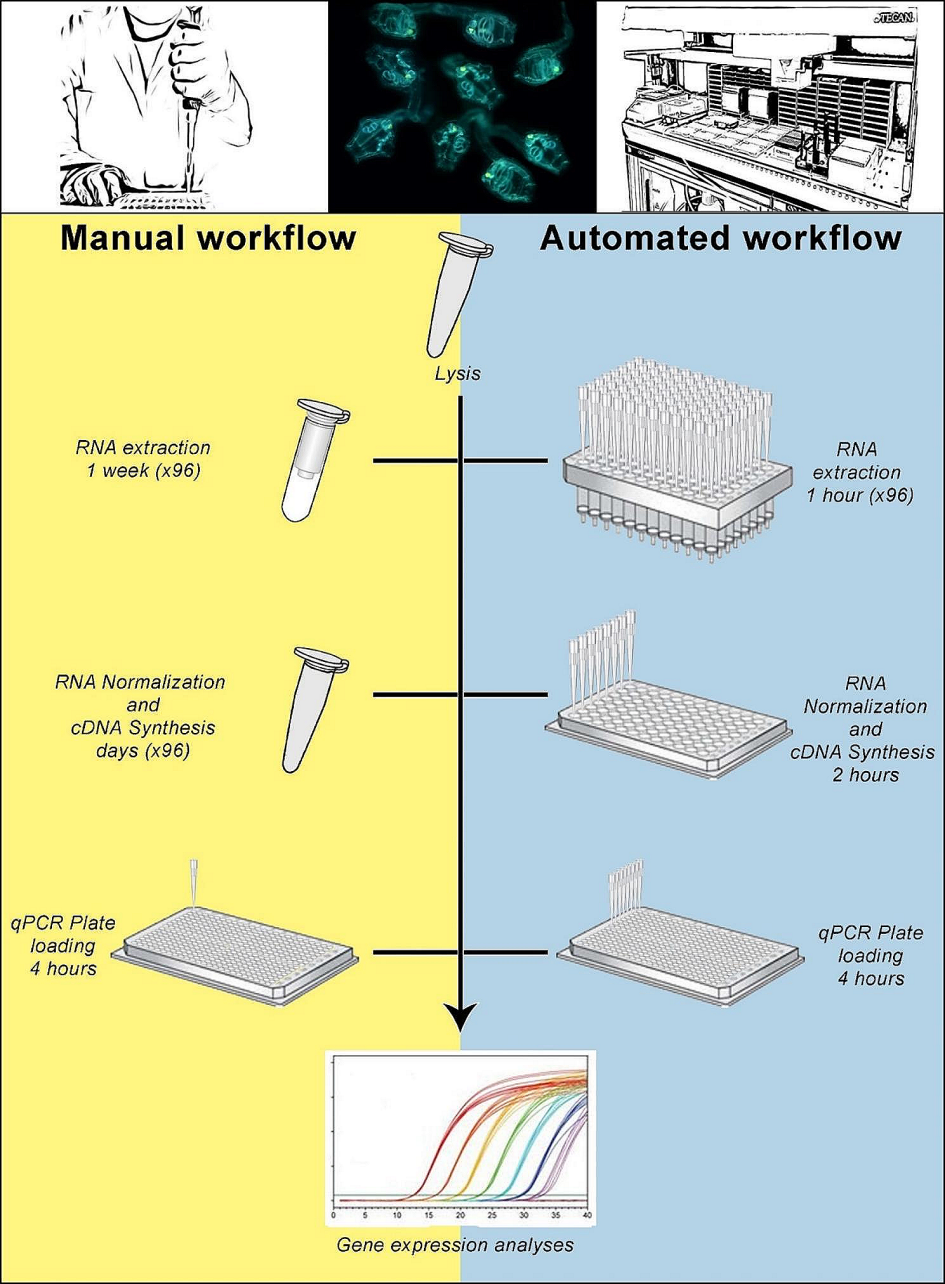
Schematic representation of manual and automated workflows for gene expression analysis. The scheme presents a comparison of the workflow and execution time required for each step between the automated and the manual procedures

*Ciona* juveniles at stage 4 of metamorphosis were treated with two microbial stimuli, specifically Pam2CSK4 at a concentration of 1 μg/ml, and zymosan at a concentration of 100 μg/ml. Treated juveniles were employed to develop and validate an automated pipeline for RNA extraction and gene expression analysis using the RT-qPCR technique. In the following sections, we illustrate the robotic platform employed and describe and discuss the results obtained at each step of the automated workflow in comparison with the results obtained using the manual workflow (Fig. 1). We also highlight the strengths and weaknesses of both procedures, providing a comprehensive analysis of their performance.

### The robotic integrated platform TECAN freedom EVO200 system

**Fig. 2:**
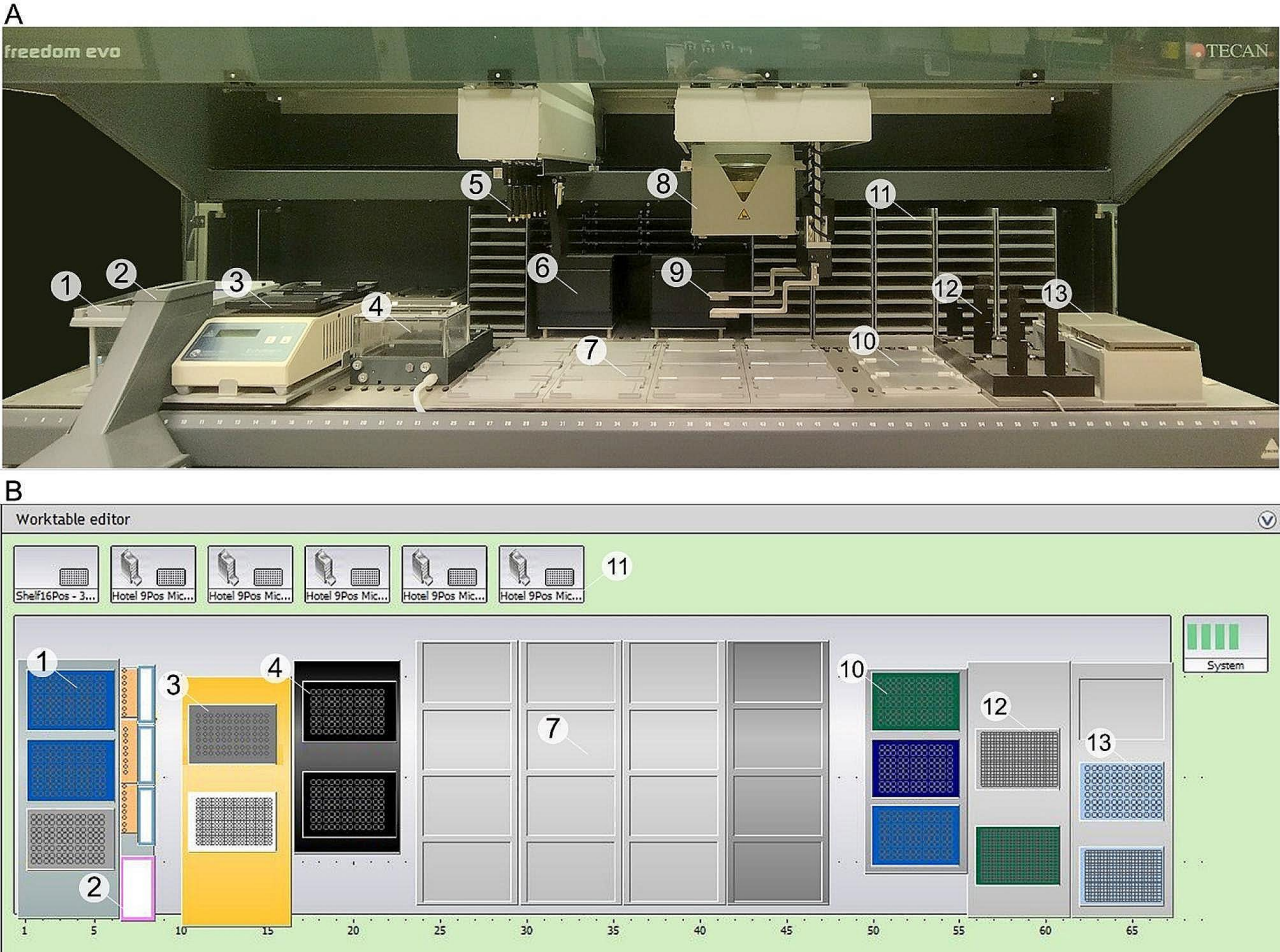
TECAN Freedom EVO200 system representation. The figure shows the liquid handling platform equipment. (**A**) Picture of the instrument supplied at the institute’s facility (Stazione Zoologica Anton Dorhn, Naples). (**B**) Scheme of the workstation. Numbers indicate the main components of the platform both (**A**) in the picture and/or (**B**) in the scheme of the workstation: 1, LiHa tips carrier (3 positions); 2, Liquid/Solid waste station (3 plus 1 positions, respectively); 3, Chilling/Heating dry baths (2 positions); 4, Vacuum Separator block (2 positions); 5, Liquid Handling Arm (LiHa); 6, Shaking Heated Incubators (2 positions); 7, Worktable (16 positions); 8, Multi-Channel Arm 96-tip pipetting head (MCA96); 9, Common Gripper Module (CGM); 10, MCA tips carrier (3 positions); 11, Plate Hotel shelf (6 positions); 12, MCA 384 tips support (2 positions); 13, MCA Head adapters

For the automated workflow we make use of the integrated robotic platform TECAN Freedom EVO200 (Fig. 2, A and B), installed at the Sequencing and Molecular Analysis Center, Stazione Zoologica Anton Dohrn, Naples, Italy. This platform, designed for high-throughput liquid handling applications, is optimized for biomolecular approaches, and equipped with mobile apparatus for solutions transfer, reaction preparation, and plate relocation. The Liquid Handling Arm (LiHa) is composed of eight independent pipetting channels that allow to aspirate, dispense, and mix solutions for reaction preparation going through several supports, ranging from microtube to cell culture multi-well plates. The Multi-Channel Arm 96-tip pipetting head (MCA96) is equipped with 96 pipetting tips that can aspirate and dispense liquids simultaneously. It is widely used for the preparation of reaction plates, extraction and purification of nucleic acids, or high-throughput molecule screening. The Common Gripper Module (CGM) is designed for the handling and transfer of the labware in all positions of the workstation for mixing, storage, or incubation processes. Moreover, the device is provided with several static tools, including chilling/heating dry baths, heated incubators with shakers, vacuum block plate base, and orbital shake mixer (Fig. 2). Orchestrating all these components makes it possible to design specific protocols for automated workflow setting.

Here we report custom protocols designed for the automated procedure for gene expression analyses, including RNA extraction and purification, RNA normalization, cDNA synthesis, and 384-well plate loading for RT-qPCR analysis.

### RNA extraction

The automated protocol for RNA extraction offers several advantages over the manual protocol. Firstly, it allows for the concurrent processing of up to 96 samples, significantly increasing the throughput compared to the manual procedure, which has limitations in sample handling and can process a lower number of samples at a time. As a result, the automated platform enables RNA extraction in approximately 1 h, whereas the manual procedure takes several days or even up to a week (as in this specific case) considering the standard procedures in the laboratory.

**Fig. 3:**
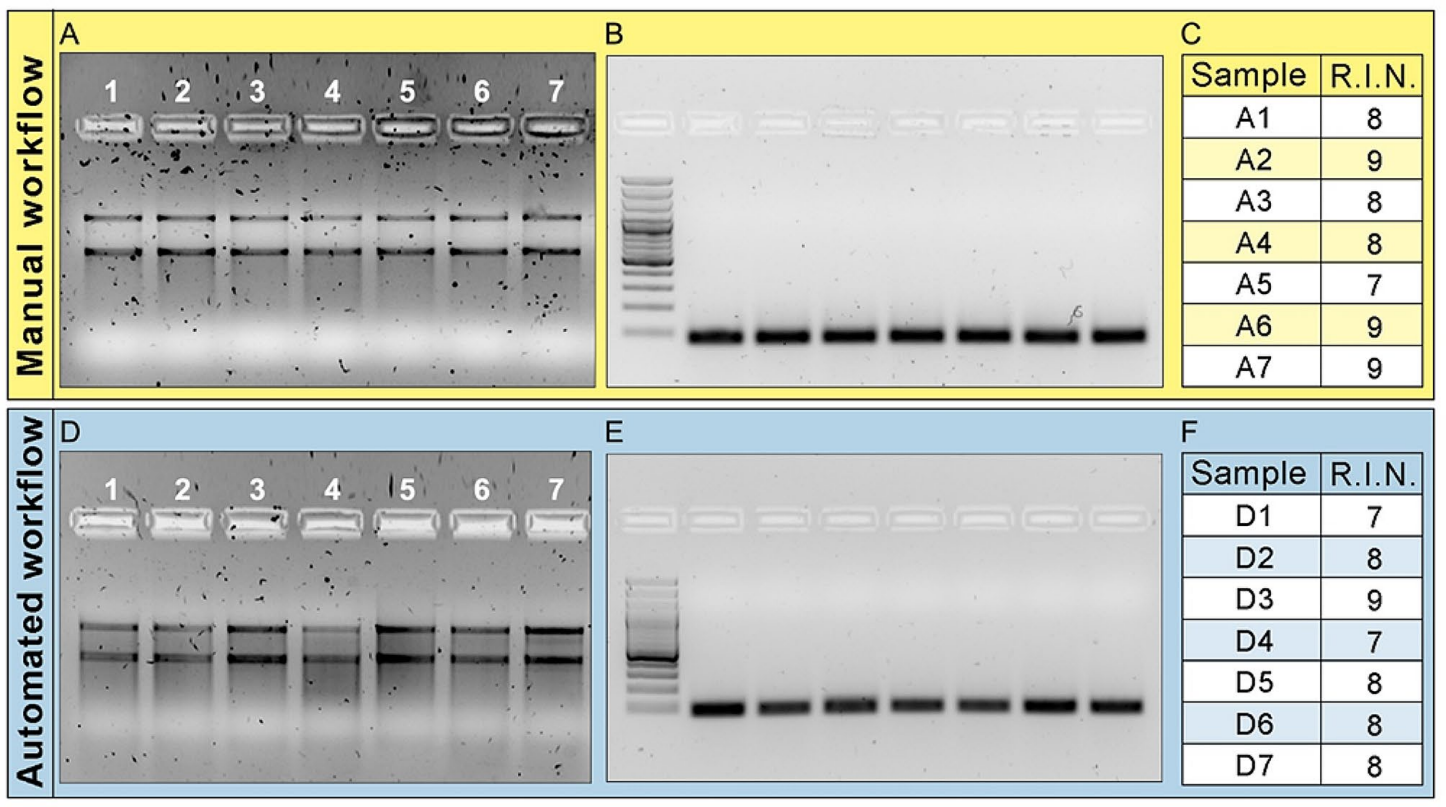
Quality control of RNA extraction and cDNA synthesis using manual and automated procedures. (**A** and **D**) Gel electrophoresis of extracted RNA using manual and automated protocol, respectively. (**B** and **E**) Gel electrophoresis of PCR amplification of *Act* coding gene using, as a template, cDNA obtained through the manual and automated procedure, respectively. (**C** and **F**) RIN values of RNA extracted using manual (samples A1-7 from picture A) and automated (samples D1-7 from picture D) protocols. The numbers from 1 to 7 indicate sample treated as discussed in method section: 1, Control; 2, Pam2CSK4 30 min; 3, Pam2CSK4 2 h; 4, Pam2CSK4 4 h; 5, zymosan 30 min; 6, zymosan 2 h; 7, zymosan 4 h. Original files of the cropped gels shown in the figure are available in Supplementary Material 5

The quality of the RNA extracted and purified using the automated protocol is comparable to that obtained with the manual protocol. This is evident from the gel electrophoresis control (Fig. 3, A and D), where the RNA bands appear intact, indicating successful extraction. Additionally, the RNA integrity number (RIN) obtained using the Agilent 2100 Bioanalyzer System confirms the high quality of the RNA extracted (Fig. 3, C and F). Specifically, the same lysate has been divided into two aliquots to be used for the automated RNA extraction and the routine manual protocol. These results support the efficacy of the automated RNA extraction protocol.

The RNA extracted through the automated procedure may have a more diluted concentration compared to the manual protocol. This difference arises from the manufacturer’s instructions, which recommend using a higher volume of Elution Solution in the automated procedure (two steps of 40 μl) compared to the manual protocol (total of 20 μl of Elution solution). Researchers should carefully consider this difference in RNA concentration for subsequent applications.

However, it is worth mentioning that a slight reduction in the final RNA yield was observed with the automated procedure compared to the manual procedure. This decrease in yield could be attributed to standard errors inherent in automated processes, which are often mitigated by operator monitoring and intervention in manual procedures. Despite this minor difference, the automated RNA extraction protocol still proves to be a reliable and efficient method.

### RNA normalization and cDNA synthesis

The cDNA synthesis step in the automated workflow includes both RNA normalization and the assembly of reaction mixes for retrotranscription. Typically, an amount of total RNA ranging from 500 ng to 1 μg is used for retrotranscription. In the automated procedure, a dedicated Work-List was designed to ensure the equalization of RNA by adding sample-specific volumes of DNase/RNase-Free water in an automated manner.

The automated cDNA synthesis process takes approximately 2 h to complete for 96 samples. In contrast, the manual protocol requires a significantly longer time, typically taking several hours or even 3–4 working days considering standard laboratory conditions and the time required for the operator that performs the steps manually.

The results obtained from the automated cDNA synthesis are comparable to those obtained from the manual procedure as evidenced by the PCR amplification of the reference gene *Act* (Fig. 3, B and E), indicating successful cDNA synthesis. To ensure the smooth progression of the automated protocol, a 10–15% surplus of reagents was considered during the calculation to account for potential variations.

Overall, the automated cDNA synthesis procedure offers significant time savings compared to the manual protocol. The quality of the synthesized cDNA is comparable and the automated process ensures reproducibility and accuracy in sample normalization and reaction mix assembly.

### RT-qPCR 384-well plate loading for gene expression analysis

The final step of the automated pipeline involved the loading of the RT-qPCR 384-well plate for gene expression analysis. To validate the reliability of the automated platform in generating gene expression data (fold changes), we analyzed seven genes associated with the *Ciona* immune system [24] using the same cDNA for both the automated and manual procedures. The oligonucleotide pairs used targeted genes such as *TLR2* (receptor), *SYK* (a coding gene acting as cofactor according to experimental design in [24]), *IRF-like* and *NF-κB* (coding for transcription factors), and cytokines including *IL17-3*, *MIF*, and *TGFβ*, which are known to be modulated by the inflammatory stimuli Pam2CSK4 and zymosan [24].

In the automated protocol, the operator’s involvement is mainly limited to the preparation of dilutions for the oligonucleotide pairs. The automated platform takes care of other tasks, including cDNA dilution to a final concentration of 5 ng/μl, assembling the reaction mixes in a 96-well plate with different combinations of oligonucleotide pairs dilutions, SYBR green reaction mix, and diluted cDNA. Finally, the platform transfers the individual reaction mixes, divided into triplicates, into the 384-well plate.

This automated protocol, from cDNA dilution to the full 384-well plate loading, takes approximately 4 h. In comparison, the manual protocol may also take a similar amount of time but may require additional resting time for the operator between the steps. Overall, the automated platform streamlines the process by minimizing manual involvement and ensuring consistent and efficient loading of the 384-well plate for gene expression analysis.

**Fig. 4:**
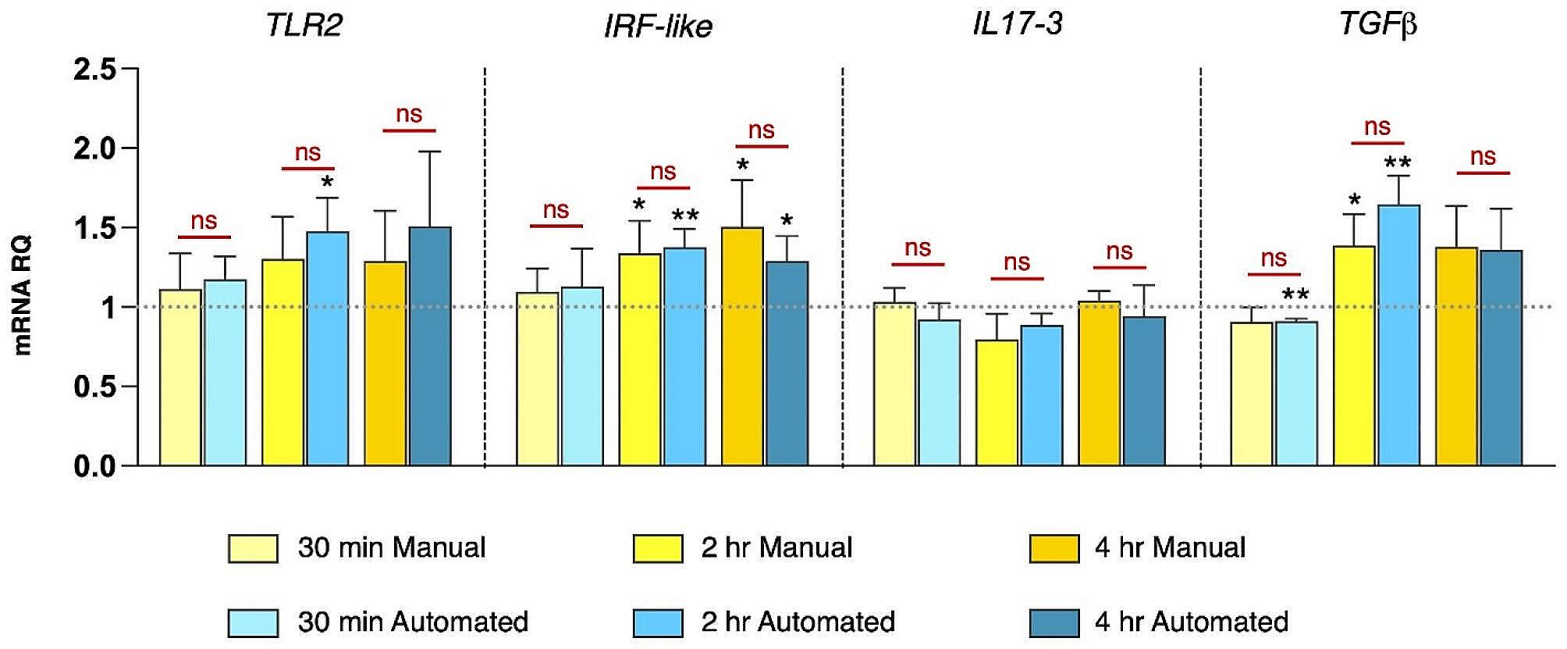
Comparison of gene expression analysis of technical replicates, through RT-qPCR, performed using automated and manual procedures. The graph reports RT-qPCR data (reported as mRNA relative quantity, mRNA RQ), performed with both manual and automated protocol of *Ciona* juveniles exposed to Pam2CSK4 (1 μg/ml), for 30 min, 2 h, and 4 h. The number of technical replicate for both automated and manual procedures was equal to 4. Graph do not show any statistical differences between the automated and manual procedures for all genes and at the same time-point treatment analyzed. ns: not statistically different (*P* > 0.05). Asterisks indicates the statistical significance between control (reported as dotted gray line) and treated samples (* *P* ≤ 0.05; ** *P* ≤ 0.01)

As a first step to confirm automation technical reliability, we have executed technical replicates (*n* = 4) for a subset of genes (i.e. *TLR*2, *IRF-like*, *IL17-3* and *TGFβ*) and one inflammatory stimulus (Pam2CSK4 at three time points) for automated and manual protocols. Comparing the results obtained by the two procedures (expressed as fold change), we did not assess any significant statistical differences (Fig. 4). Moreover, analyzing gene modulation patterns we observed a correspondence in the statistical significance between the two procedures, showing a major accuracy in the automated procedure highlighted by a stronger significance level compared to the manual approach (Fig. 4).

**Fig. 5:**
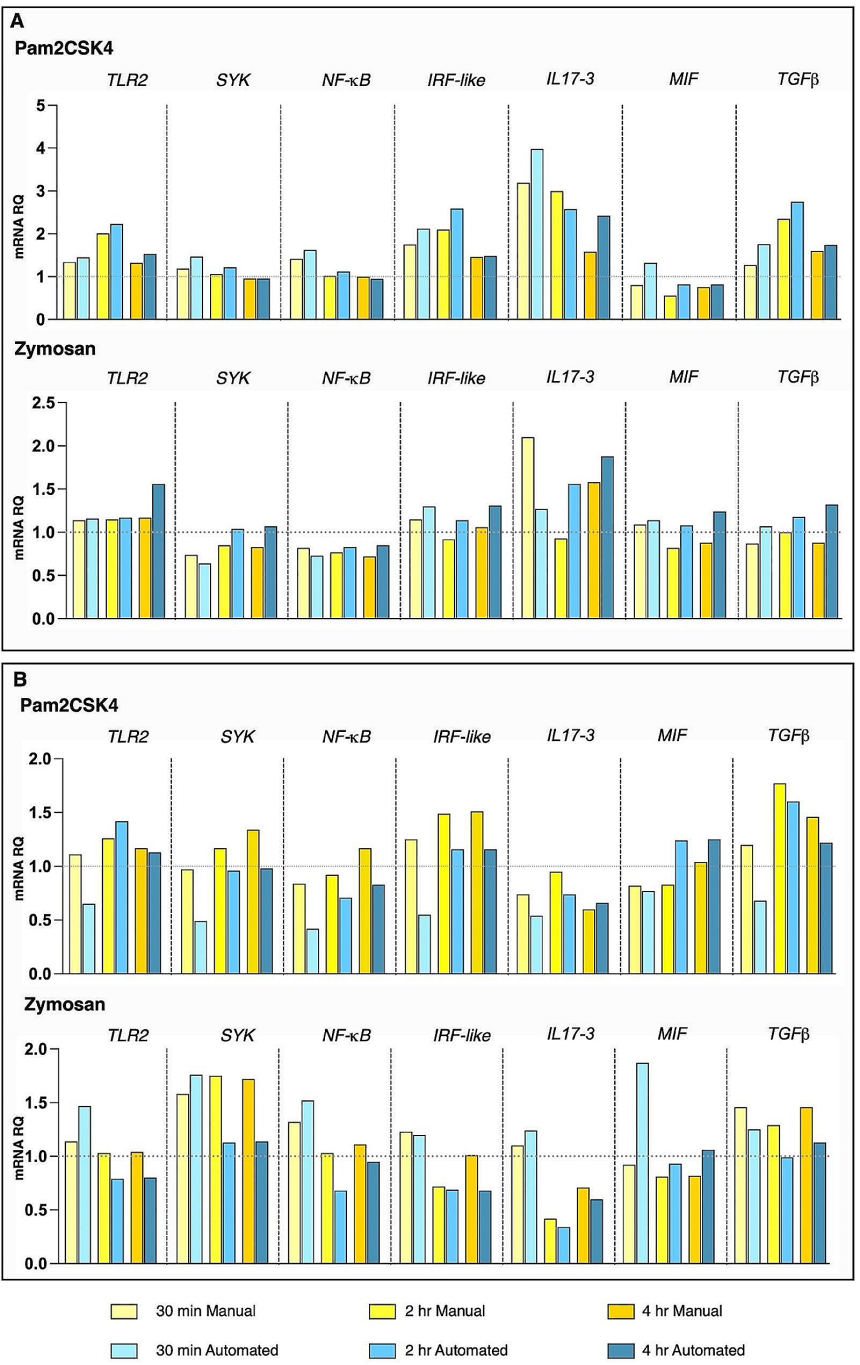
Comparison of gene expression analysis in two biological replicates by RT-qPCR, performed using automated and manual procedures. The graphs report the gene expression, represented as mRNA relative quantity (mRNA RQ), of Ciona juveniles exposed to Pam2CSK4 (1 μg/ml) or zymosan (100 μg/ml), for 30 min, 2 h, and 4 h. (**A**), “Biological replicate 1”, resulting in a more effective response to the microbial stimuli treatment, reflects a clearer comparison between the two procedures. In contrast (**B**), less responsive animals (“biological replicate 2”) display higher variability in gene expression detection

Later, in order to analyze the biological response related to the treatments we analysed the expression of the full set of genes in response to the two inflammatory stimuli (reported above), for the evaluation of putative differences ascribed to the two workflows. We observed that when biological samples exhibit strong transcriptional modulation (Fig. 5A, reported as “biological replicate 1”), characterized by fold changes above 1.5/1.8 or below 0.5/0.6, following inflammatory stimulus treatment, the results of gene expression analysis obtained with the automated protocol are consistent with those obtained using the manual 384-well plate loading (Fig, 5. A). This indicates that the automated protocol accurately detects significant changes in gene expression.

However, when samples exhibit slight modulation of gene expression (between 0.7 and 1.4) (Fig. 5, B, reported as “biological replicate 2”), which can occur due to natural biological diversity, we detected a certain degree of variability in the detection of gene expression between the two protocols (Fig, 5, A and C). This result highlights the importance of testing multiple biological replicates. The automated workflow becomes particularly useful in such scenarios by improving operational efficiency in processing a large number of replicates.

It is worth noting that occasionally, a slight difference in Ct value may be observed within a technical triplicate of the automated protocol. However, these differences can be resolved by performing three technical replicates. Additionally, it is recommended to consider an excess of reagents (10–15%) in the calculation of reaction mixtures to account for the repeated pipetting operations and the potential waste of reagents during the reaction assembly process.

Overall, the automated workflow provides a valuable tool for handling large-scale experiments and is particularly advantageous when dealing with samples that exhibit significant transcriptional modulation.

## Conclusions

High-throughput fully automated platforms have revolutionized laboratory protocols across several research fields. These platforms have been successfully utilized in chemistry, biochemistry, microbiology, and biomedical research, enabling automated drug discovery [30], proteomics sample preparation [31], microbial monoclonal cultivation [32], and biomedical applications [33].

In this study, for the first time, we evaluated the accuracy of molecular biology experimental procedures using a high-throughput fully automated platform. The sequence of tasks, activities, and processes encompassed RNA extraction and purification, cDNA synthesis, and RT-qPCR plate loading to analyze gene expression in a whole organism exposed to microbial stimuli. The results obtained from the automated procedures were comparable to those obtained using manual protocols for each step of the workflow, although the automated approach shows a major accuracy of the technical replicates and results more advantageous in terms of execution time, particularly during the initial steps such as RNA extraction and purification, normalization, and cDNA retrotranscription.

During the automated procedures, a surplus of reagents was utilized, particularly for viscous solutions such as SYBR reagent. However, this excess was justified by the reduction in execution times and the ability to process a large number of samples with consistent accuracy. The technology demonstrated high adaptability to different requirements and ensured data reproducibility while significantly saving time.

This study highlights the broad application potential of high-throughput automated platforms. It opens up new possibilities for large-scale compound screening, the use of non-conventional model systems that can facilitate translational approaches, and other advanced molecular biology applications. The automated workflow provides researchers with an efficient and reliable tool to handle complex experiments and generate high-quality data. High-throughput toxicological or pharmacological screening using gene expression profiling is now well within reach.

## Methods

### Ethics statement and sample preparation

The research described herein was performed on *Ciona* specimens collected in the Fusaro lagoon (Naples, Italy), in locations that are not privately owned nor protected in any way, according to the authorization of Marina Mercantile (DPR 1639/68, 09/19/1980, confirmed on 01/10/2000). The study was carried out in strict accordance with European (Directive 2010/63) and Italian legislation for the care and use of animals for scientific purposes (Legislative Decree n. 26/2014).

*Ciona* specimens were maintained in clean seawater with aeration, temperature control, and properly fed. To stimulate gamete maturation, animals were exposed to constant light. In vitro fertilization was conducted by surgically collecting eggs and spermatozoa from the gonoducts of different animals, as previously described [19] with the exception that 0.22 μM filtered seawater was used and no sterilization step was performed. At 18 h post-fertilization, during the swimming tadpole larval stage (as described in [34]), approximately 1500 individuals were transferred to a 60 mm plate, where they underwent metamorphosis. *Ciona* juveniles at stage 4 of metamorphosis (5 days post fertilization), were treated for 30 min (min), 2 and 4 h (hr) with two microbial stimuli: the bacterial diacyl-lipoprotein Pam2CSK4 (InvivoGen #tlrl-pm2s-1) at the concentration of 1 μg/ml, and fungal zymosan (InvivoGen #tlrl-zyn) at the concentration of 100 μg/ml. Then, they were collected and stored at -20 °C as reported in *Liberti et al. 2023* [24]. For each experiment, samples were randomly chosen and three biological replicates were performed.

Before proceeding with either the automated or manual RNA extraction protocol, the plates containing the treated juveniles were slowly allowed to thaw. After adding 400 μl of lysis buffer (contained in the RNA extraction kit - Invitrogen #AM1812) all over the plate, animals were detached by scraping them using a flat blade cell lifter. They were then collected and transferred to a 1.5 ml microtube. To mechanically break them, an ultra sonicator was used (Branson) for 15 s (sec) at 20% of maximum power. The lysate was then divided into two equal parts, with 50% of the lysate allocated for the manual RNA extraction protocol and the remaining 50% for the automated workflow. The following sections provide a detailed description of each protocol.

### Manual protocols for RNA extraction, cDNA synthesis, and gene expression analysis

Manual RNA extraction and purification, cDNA synthesis, and RT-qPCR were performed as previously described [24]. Briefly, total RNA was extracted using RNAqueous™-Micro Total RNA Isolation Kit (Invitrogen #AM193), following the manufacturer’s instructions. The extracted RNA was eluted using a 20 μl elution buffer. To eliminate any DNA contamination, the DNaseI step was performed, as outlined in the manufacturer’s procedure. RNA quality and quantity were evaluated through 2% agar gel electrophoresis and NanoDrop spectrophotometer (Themo-Fisher) reading. Further RNA quality control was performed using the Eukaryote Total RNA Nano kit (#5067 − 1511, Agilent Technologies) on the 2100 Bioanalyzer System (G2939A, Agilent Technologies). Single-stranded cDNA was synthesized from 1 μg of total RNA by employing a QuantiTect Reverse Transcription kit (Qiagen #205,311). According to the manufacturer’s recommendation, RT-qPCR was conducted using the Power Track™ SYBR™ Green Master Mix (Applied Biosystems #46,109). The reaction mixture consisted of 0.4 μM for each primer and 5 ng of cDNA per reaction. The primer sequences of the examined genes, including *Toll-like receptor 2* (*TLR2*), *Tyrosine-protein kinase* (*SYK*), *Interferon regulatory factor-like* (*IRF-like*), *Nuclear factor kappa B* (*NF-κB*), *Interleukin 17 − 3* (*IL17-3*), *Macrophage migration inhibitory factor* (*MIF*) and *Transforming growth factor beta* (*TGFβ*) along with *Cytoskeletal actin* (*Act*) (for internal standardization), are provided in [24]. The RT-qPCR program used for the experiments followed the instructions provided by the manufacturer. A denaturation step at 95 °C for 2 min, 40 amplification cycles (95 °C for 5 s and 60 °C for 30 s), and a Melt Curve step (95 °C for 15 s, 60 °C for 1 min and 95 °C for 15 s) were employed. Reactions, for each sample, were performed in triplicate. To calculate mRNA expression level (mRNA RQ) relative to the control sample, data were analyzed with QuantStudio™ Design & Analysis software v1.5.2, (Life Technologies) and quantified with the comparative *C*_*t*_ method (2^−ΔΔ*Ct*^) based on cycle threshold (Ct) values [24]. Data were expressed as fold change of treated vs. untreated samples. Graphical representations and the statistical analysis (unpaired parametric t-test) on the technical replicates (*n* = 4) have been performed using GraphPad PRISM software, version 10.1.0.

### Automated protocols

The automated workflow utilized the TECAN Freedom EVO200 system, an integrated robotic platform specifically optimized for high-throughput liquid handling applications. Custom protocols were designed for each automated application, including RNA extraction and purification, RNA normalization, cDNA synthesis, and 384-well plate loading for RT-qPCR analysis.

#### RNA extraction

The lysed samples were placed in a 96-well plate and subjected to RNA extraction using the RNAqueous-96 Automated Kit (Invitrogen #AM1812). Using the Multi-Channel Arm 96-tip pipetting head (MCA96), 200 μl of 100% ethanol were added to each well containing 200 μl of *Ciona* juveniles’ lysate. The mixture was thoroughly mixed and transferred to a filter plate. A vacuum of 120 s was applied using the equipped Te-VacS (vacuum separator) to draw the solution through the filter. Next, the samples were washed with 300 μl of Wash Solution (WS), followed by another vacuum step. For the DNase treatment, 20 μl DNaseI were directly added to the filter of each well in the filter plate. After a digestion step of 15 min at room temperature, the samples were washed with 200 μl of Rebinding Mix. Following a 60-sec pause, a vacuum step of 60 s was applied. Then, two additional washes were performed with 200 μl of WS added to each filter well, followed by vacuum steps of 60 s and 5 min, respectively, to dry the filter plate. After mechanically placing the filter on the collecting 96-well plate using the robot arm CGM, the MCA96 head was used for a double step of RNA elution with 40 μl nuclease-free water. Finally, 10 μl of the extracted RNA was transferred to a new 96-well plate for routine quality-quantity control check, while the original collection plate was stored at -80 °C. To assess RNA quality and quantity, the extracted RNA was evaluated using 2% agar gel electrophoresis and NanoDrop spectrophotometer (Themo-Fisher) reading, following the same methods as described in the manual protocol. Further RNA quality control was performed using the Eukaryote Total RNA Nano kit (#5067 − 1511, Agilent Technologies) on the 2100 Bioanalyzer System (G2939A, Agilent Technologies). A more detailed protocol can be found in Supplementary Material 1.

#### RNA normalization

To ensure equalization of the RNA template for the subsequent cDNA synthesis, the LiHa was utilized. To facilitate this process, a comprehensive Work-List was developed, which consisted of text files containing all the pipetting instructions, including the source and destination positions, as well as the volumes to be pipetted. For each sample, specific volumes of RNA and nuclease-free water were pipetted into a 96-well plate to achieve a final concentration of 500 ng of RNA in a total volume of 12 μl. This plate was then utilized in the subsequent cDNA synthesis protocol. For further details, please refer to Supplementary Material 2 for the comprehensive protocol.

#### cDNA synthesis

The cDNA preparation process, using QuantiTect Reverse Transcription kit (Qiagen #205,311), followed two subsequent steps with two different MasterMixes, as described in the manufacturer’s instructions. The first MasterMix was used for genomic DNA (gDNA) erasure and the second MasterMix for cDNA retrotranscription. For the gDNA eraser step, the LiHa was employed to add 2 μl of enzyme-buffer to each sample. The samples were then incubated for 2 min at 42 °C. Subsequently, using a similar command, 6 μl of reverse-transcription Master Mix were pipetted into the sample plate and incubated first for 15 min at 42 °C and then for 3 min at 95 °C to inactivate the reverse transcriptase enzyme. The newly synthesized cDNA samples could be stored or used for the RT-qPCR analyses. For a more detailed protocol, see Supplementary Material 3.

#### RT-qPCR 384-well plate loading

The protocol for assembling the RT-qPCR plates (384-well) using the LiHa involved the use of loop functions. The process started with the dilution step of cDNA to the final concentration of 5 ng/μl, followed by the assembly of the reaction mix for each combination of specific oligonucleotide pairs and cDNA in a 96-well plate. The reaction mix for each well in the 96-well plate, corresponding to triplicate volume, was prepared by sequentially adding oligonucleotide pairs at a final concentration of 0.4 μM each, Power Track™ SYBR™ Green Master Mix (Applied Biosystems #46,109), and diluted cDNA. The final step involved a loop operation where each reaction mix was pipetted and dispensed into three consecutive wells of the 384-well plate (triplicates), resulting in a final volume of 10 μl of reaction mix per replica. The loaded 384-well plate was then ready for processing in the real-time PCR system for gene expression profile analysis. A more detailed protocol is available in Supplementary Material 4.

To minimize potential differences arising from procedural and experimental inaccuracies throughout the comparative manual-automated gene expression analyses, the cDNA obtained from the same RNA retrotranscription was used. In order to conduct a comprehensive comparative examination, the gene expression analyses were performed by loading the same experimental set (consisting of 4 experimental conditions and 5 genes, and 7 experimental conditions and 8 genes for technical and biological replicates, respectively) on the same 384-well plate for both the manual and automated procedures.

### Electronic supplementary material

Below is the link to the electronic supplementary material.

**Supplementary Material 1: Supplementary Material 1.** Automated workflow RNA Extraction script

**Supplementary Material 2: Supplementary Material 2.** Automated workflow RNA normalization script

**Supplementary Material 3: Supplementary Material 3.** Automated workflow cDNA synthesis script

**Supplementary Material 4: Supplementary Material 4.** Automated workflow RT-qPCR 384-well plate loading

**Supplementary Material 5: Supplementary Material 5.** Full-length gel electrophoresis reported in Figure 3 “Quality control of RNA extraction and cDNA synthesis using manual and automated procedures”. (A and B) Full-length of the RNA cropped gel reported in Panel A and Panel D of the Figure 3, respectively. (C and D) Full-length of the PCR cropped gel reported in Panel B and in Panel E of Figure 3, respectively, that correspond to the lower part of the full-length gels

## Data Availability

All data generated or analyzed during the current study are included in this published article and its supplementary information files. All further data will be provided by the corresponding authors at any time upon request.

## Abbreviations

Act: Cytoskeletal actin
CGM: Common Gripper Module
cDNA: Complementary DNA
Ct: Cycle threshold
DNA: Deoxyribonucleic acid
gDNA: Genomic DNA
Hr: Hour
IL17-3: Interleukin 17-3
IRF-like: Interferon regulatory factor-like
LiHa: Liquid Handling Arm
MCA96: Multi-Channel Arm 96-tip pipetting head
MIF: Macrophage migration inhibitory factor
Min: Minute
NF-κB: Nuclear factor kappa B
PCR: Polymerase chain reaction
RIN: RNA integrity number
RNA: Ribonucleic Acid
RT-qPCR: Reverse transcription-quantitative PCR
Sec: Second
SYK: Tyrosine-protein kinase
TGFβ: Transforming growth factor beta
TLR2: Toll-like receptor 2
WS: Wash Solution
