# Supplementary material for: Reaping the benefits of liquid handlers for high-throughput gene expression profiling in a marine model invertebrate
Source: BMC Biotechnol. 2024 Jan 19;24:4. doi: 10.1186/s12896-024-00831-y (PMC10799371; doi:10.1186/s12896-024-00831-y)

Supplementary Material 1. Automated workflow RNA Extraction script.

Step	Command	Parameters
1	User Prompt	"Pump ON"<br>sound : no
2	User Prompt	"To Warm DEPC Water (65°C)"<br>sound : no
3	Wash Tips	1-8 30 + 40 ml
4	Group	Variables
5	Set Variable	DNAse<br>"DNAse DONE (0) or TO DO (1)?"<br>"DNAse DONE (0) or TO DO (1)?", 0 - 1
6	Set Variable	PreHeat<br>"Warm YES (0) or NOT (1)?"<br>"Warm YES (0) or NOT (1)?", 0 - 1
7	Set Variable	DNAse_Orig<br>"DNAse EPPENDORF (0) or PLATE (1)?"<br>"DNAse EPPENDORF (0) or PLATE (1)?", 0 - 1
8	Set Variable	DNAse_37°C<br>"DNAsi 37°C YES (0) NO (1)?"<br>"DNAsi 37°C YES (0) NO (1)?", 0 - 1
9	Group End	Variables
10	If - Then	DNAse_37°C = 0
11	User Prompt	"DNAse to 37°C"<br>sound : no
12	Else	
13	End If	
14	Group	Add Ethanol (Step 1-b-ii)
15	Get Head Adapter	Grid 62; Site: 2 (Adapter 96 DiTi 4to1 MCA384)
16	Get DiTis	Grid 50; Site: 1 (DiTi 200ul SBS MCA96)<br>Adapter 96 DiTi 4to1 MCA384
17	Aspirate	100 µl Ethanol 85%_Plate<br>"Ethanol100" (Col. 1, Rows 1,3,5,7,9,11,13,15)
18	Dispense	100 µl Ethanol 85%_Plate<br>"RNA Lysate" (Col. 1, Rows 1-8)
19	Mix	180 µl >> Water free dispense lv <<<br>"RNA Lysate" (Col. 1, Rows 1-8)
20	Begin Loop	2 times "Filt"
21	Aspirate	150 µl Water free dispense<br>"RNA Lysate" (Col. 1, Rows 1-8)
22	Dispense	150 µl Water free dispense<br>"Filtr Pl Ciona" (Col. 1, Rows 1-8)

23	End Loop	"Filt"
24	Drop DiTis	Back to Source
25	Comment	PUMP ON
26	Command	"O2SSO4,1"<br>wait
27	Start Timer	1
28	Wait for Timer	Timer 1 : 180 sec
29	Comment	PUMP OFF
30	Command	"O2SSO4,0"<br>wait
31	Group End	Add Ethanol (Step 1-b-ii)
32	Group	DNAse
33	If - Then	DNAse = 0
34	Get DiTis	Grid 50; Site: 2 (DiTi 200ul SBS MCA96)<br>Adapter 96 DiTi 4to1 MCA384
35	Begin Loop	2 times "Wash"
36	Aspirate	150 µl Water free dispense<br>"WS" (Col. 1, Rows 1,3,5,7,9,11,13,15)
37	Dispense	150 µl Water free dispense<br>"Filtr Pl Giona" (Col. 1, Rows 1-8)
38	End Loop	"Wash"
39	Drop DiTis	Back to Source
40	Comment	PUMP ON
41	Command	"O2SSO4,1"<br>wait
42	Start Timer	2
43	Wait for Timer	Timer 2 : 120 sec
44	Comment	PUMP OFF
45	Command	"O2SSO4,0"<br>wait
46	If - Then	DNAse_Orig = 0

47	User Prompt	"Place DNAse (Eppendorf)"<br>sound : no
48	Begin Loop	3 times "DNAseDisp"
49	Get DiTis	1-8 DiTi 50ul LiHa
50	Aspirate	1 20 µl Water free dispense New "DNAseT" (Col. 1, Row 4)
51	Aspirate	2 20 µl Water free dispense New "DNAseT" (Col. 1, Row 4)
52	Aspirate	3 20 µl Water free dispense New "DNAseT" (Col. 1, Row 4)
53	Aspirate	4 20 µl Water free dispense New "DNAseT" (Col. 1, Row 4)
54	Aspirate	5 20 µl Water free dispense New "DNAseT" (Col. 1, Row 4)
55	Aspirate	6 20 µl Water free dispense New "DNAseT" (Col. 1, Row 4)
56	Aspirate	7 20 µl Water free dispense New "DNAseT" (Col. 1, Row 4)
57	Aspirate	8 20 µl Water free dispense New "DNAseT" (Col. 1, Row 4)
58	Dispense	1-8 20 µl Water free dispense New "Filtr Pl Ciona" (Col. 1, Rows 1-8) , 1 option
59	Drop DiTis	1-8 Washstation 2Grid DiTi Waste
60	End Loop	"DNAseDisp"
61	Start Timer	3
62	Wait for Timer	Timer 3 : 900 sec
63	Else	
64	Get DiTis	Grid 50; Site: 1 (DiTi 200ul SBS MCA96)<br>Adapter 96 DiTi 4to1 MCA384
65	Aspirate	20 µl Water MCA96 "DNAseP" (Col. 1, Rows 1-8)
66	Dispense	20 µl Water MCA96 "Filtr Pl Ciona" (Col. 1, Rows 1-8)
67	Drop DiTis	Back to Source
68	Start Timer	3
69	Wait for Timer	Timer 3 : 900 sec
70	End If	

71	Else	
72	End If	
73	Group End	DNAse
74	Group	Pre-Heat Elution
75	If - Then	PreHeat = 0
76	Else	
77	End If	
78	Group End	Pre-Heat Elution
79	Group	Rebinding Mix
80	Get DiTis	Grid 50; Site: 2 (DiTi 200ul SBS MCA96)<br>Adapter 96 DiTi 4to1 MCA384
81	Begin Loop	2 times "BindMiX"
82	Aspirate	100 µl Water free dispense<br>"RebindingMix" (Col. 1, Rows 1,3,5,7,9,11,13,15)
83	Dispense	100 µl Water free dispense<br>"Filtr Pl Ciona" (Col. 1, Rows 1-8)
84	End Loop	"BindMiX"
85	Drop DiTis	Back to Source
86	Start Timer	4
87	Wait for Timer	Timer 4 : 60 sec
88	Comment	PUMPON
89	Command	"O2SSO4,1"<br>wait
90	Start Timer	5
91	Wait for Timer	Timer 5 : 60 sec
92	Comment	PUMPOFF
93	Command	"O2SSO4,0"<br>wait
94	Group End	Rebinding Mix

No.	Command	Parameters
95	Group	Wash Solution
96	Get DiTis	Grid 50; Site: 2 (DiTi 200ul SBS MCA96)<br>Adapter 96 DiTi 4to1 MCA384
97	Begin Loop	2 times "WashSolT"
98	Begin Loop	2 times "WashSol"
99	Aspirate	100 µl Water free dispense<br>"WS" (Col. 1, Rows 1,3,5,7,9,11,13,15)
100	Dispense	100 µl Water free dispense<br>"Filtr Pl Ciona" (Col. 1, Rows 1-8)
101	End Loop	"WashSol"
102	Comment	PUMP ON
103	Command	"O2SSO4,1"<br>wait
104	Start Timer	6
105	Wait for Timer	Timer 6 : 60 sec
106	Comment	PUMP OFF
107	Command	"O2SSO4,0"<br>wait
108	End Loop	"WashSolT"
109	Comment	PUMP ON
110	Command	"O2SSO4,1"<br>wait
111	Start Timer	6
112	Wait for Timer	Timer 6 : 300 sec
113	Comment	PUMP OFF
114	Command	"O2SSO4,0"<br>wait
115	Drop DiTis	Back to Source
116	Group End	Wash Solution
117	Group	Elution Solution
118	If - Then	PreHeat = 0

119	Comment	Move Elution Plate from incubator to WT
120	Else	
121	End If	
122	User Prompt	"Place pre-heated DEPC Water (65°C)"<br>sound : no
123	User Prompt	"Place Collection Plate + Support"<br>sound : no
124	Get DiTis	Grid 50; Site: 3 (DiTi 50ul SBS MCA96)<br>Adapter 96 DiTi 4to1 MCA384
125	Aspirate	40 µl Water free dispense New<br>"Hot Elution Buffer H2O" (Col. 1, Rows 1,3,5,7,9,11,13,15)
126	Dispense	40 µl >> Water free dispense New <<<br>"Filtr Pl Ciona" (Col. 1, Rows 1-8)
127	Drop DiTis	Grid 50; Site: 3 (DiTi 50ul SBS MCA96)<br>Adapter 96 DiTi 4to1 MCA384
128	Start Timer	7
129	Wait for Timer	Timer 7 : 30 sec
130	Comment	PUMP ON
131	Command	"O2SSO4,1"<br>wait
132	Start Timer	8
133	Wait for Timer	Timer 8 : 150 sec
134	Comment	PUMP OFF
135	Command	"O2SSO4,0"<br>wait
136	User Prompt	"Place Plate in Pos1, Row 1"<br>sound : no
137	Get DiTis	Grid 50; Site: 3 (DiTi 50ul SBS MCA96)<br>Adapter 96 DiTi 4to1 MCA384
138	Aspirate	40 µl >> Water free dispense New <<<br>"Eluate 1" (Col. 1, Rows 1-8)
139	User Prompt	"Place the plate in Filter Position"<br>sound : no
140	Dispense	40 µl Water free dispense New<br>"Filtr Pl Ciona" (Col. 1, Rows 1-8)
141	Drop DiTis	Grid 50; Site: 3 (DiTi 50ul SBS MCA96)<br>Adapter 96 DiTi 4to1 MCA384
142	Start Timer	9

143	Wait for Timer	Timer 9 : 30 sec
144	Comment	PUMP ON
145	Command	"O2SSO4,1"<br>wait
146	Start Timer	10
147	Wait for Timer	Timer 10 : 150 sec
148	Comment	PUMP OFF
149	Command	"O2SSO4,0"<br>wait
150	Group End	Elution Solution
151	User Prompt	"Collection Plate READY - Move it in position 3<br>"<br>sound : no
152	Get DiTis	Grid 50; Site: 3 (DiTi 50ul SBS MCA96)<br>Adapter 96 DiTi 4to1 MCA384
153	Aspirate	5 µl Water free dispense New<br>"RNA Eluito Final" (Col. 1, Rows 1-8)
154	Dispense	5 µl Water free dispense Iv<br>"1/3 RNA Check" (Col. 1, Rows 1-8)
155	Drop DiTis	Grid 50; Site: 3 (DiTi 50ul SBS MCA96)<br>Adapter 96 DiTi 4to1 MCA384
156	Drop Head Adapter	Grid 62; Site: 2 (Adapter 96 DiTi 4to1 MCA384)

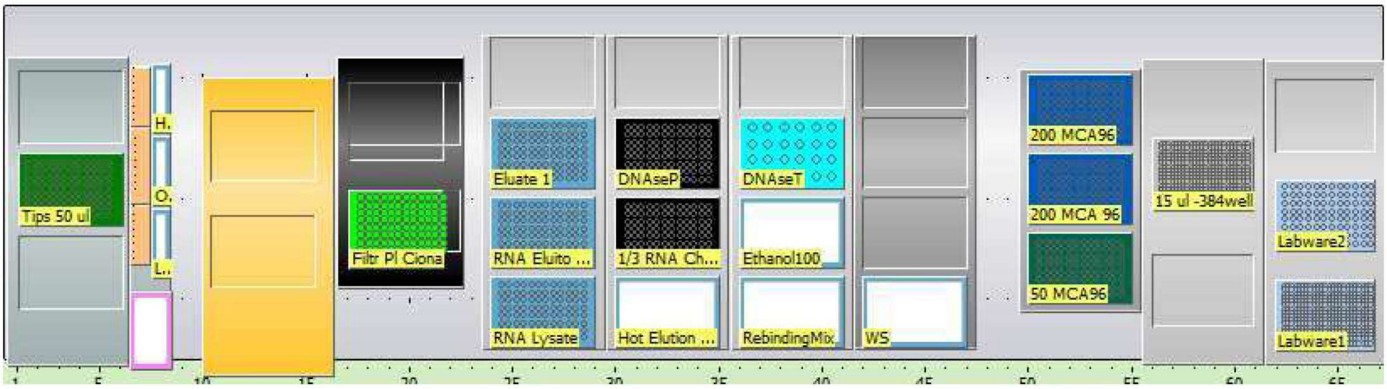

Supplement: Supplementary file 1 — Supplementary Material 1: Supplementary Material 1. Automated workflow RNA Extraction script [file 12896_2024_831_MOESM1_ESM.pdf]
