# Supplementary material for: Reaping the benefits of liquid handlers for high-throughput gene expression profiling in a marine model invertebrate
Source: BMC Biotechnol. 2024 Jan 19;24:4. doi: 10.1186/s12896-024-00831-y (PMC10799371; doi:10.1186/s12896-024-00831-y)

Supplementary Material 2. Automated workflow RNA normalization script.

Script : RNA Normalization
User : Giovanni Annona

5:46:51 PM 1/9/2023

1	Wash Tips	30 + 40 ml
2	Set DiTi position	DiTi 50ul LiHa<br>Grid : 1, Site : 2, First position in labware : 1
3	Group	Water
4	Worklist Import	C:\Users\Administrator\Desktop\Ivan\WorkList_Ivan\csv\RNA_Normalization_Water_Ciona_07.12.22.csv<br>C:\Users\Administrator\Desktop\Ivan\WorkList_Ivan\gwl\RNA_Normalization_Water_Ciona_07.12.22.gwl
5	Worklist	Load Worklist "C:\U sers\Administrator\ Desktop\Ivan\WorkLi st_Ivan\gwl\RNA_Nor malization_Water_Ci ona_07.12.22.gwl"
6	Worklist	Execute loaded worklist(s)
7	Group End	Water
8	Group	RNA
9	Worklist Import	C:\Users\Administrator\Desktop\Ivan\WorkList_Ivan\csv\RNA_Normalization_RNA_Ciona_07.12.22.csv<br>C:\Users\Administrator\Desktop\Ivan\WorkList_Ivan\gwl\RNA_Normalization_RNA_Ciona_07.12.22.gwl
10	Worklist	Load Worklist "C:\U sers\Administrator\ Desktop\Ivan\WorkLi st_Ivan\gwl\RNA_Nor malization_RNA_Cion a_07.12.22.gwl"
11	Worklist	Execute loaded worklist(s)
12	Group End	RNA

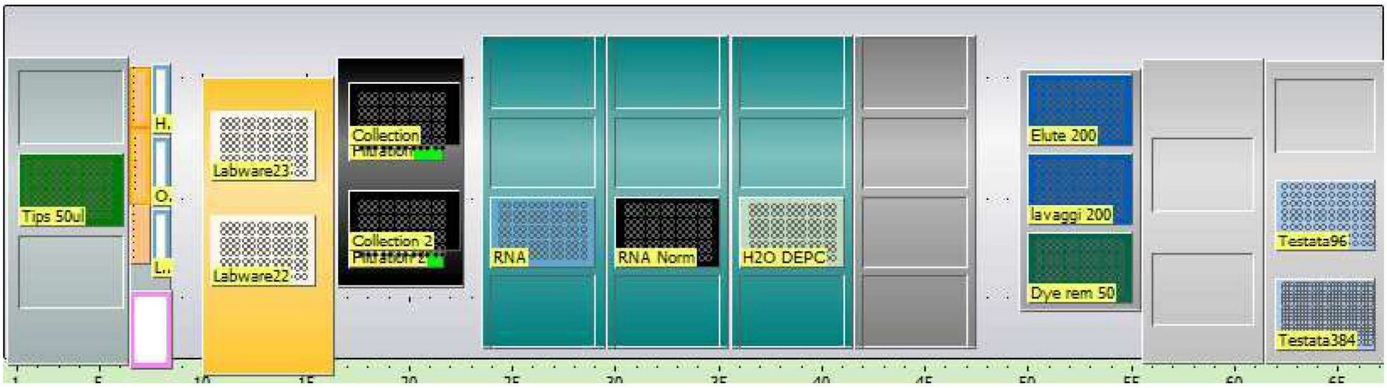

Supplement: Supplementary file 2 — Supplementary Material 2: Supplementary Material 2. Automated workflow RNA normalization script [file 12896_2024_831_MOESM2_ESM.pdf]
