# Supplementary material for: Reaping the benefits of liquid handlers for high-throughput gene expression profiling in a marine model invertebrate
Source: BMC Biotechnol. 2024 Jan 19;24:4. doi: 10.1186/s12896-024-00831-y (PMC10799371; doi:10.1186/s12896-024-00831-y)

Supplementary Material 3. Automated workflow cDNA synthesis script.

Script : cDNA Retrotranscription
User : Giovanni Annona

Step	Command	Tips	Parameters
1	Wash Tips	1-8	30 + 40 ml
2	Begin Loop		3 times "gDNA_Eraser"
3	Get DiTis	1-8	DiTi 50ul LiHa
4	Aspirate	1	2 µl Water free dispense Iv "gDNA - MM" (Col. 1, Row 4)
5	Aspirate	2	2 µl Water free dispense Iv "gDNA - MM" (Col. 1, Row 4)
6	Aspirate	3	2 µl Water free dispense Iv "gDNA - MM" (Col. 1, Row 4)
7	Aspirate	4	2 µl Water free dispense Iv "gDNA - MM" (Col. 1, Row 4)
8	Aspirate	5	2 µl Water free dispense Iv "gDNA - MM" (Col. 1, Row 4)
9	Aspirate	6	2 µl Water free dispense Iv "gDNA - MM" (Col. 1, Row 4)
10	Aspirate	7	2 µl Water free dispense Iv "gDNA - MM" (Col. 1, Row 4)
11	Aspirate	8	2 µl Water free dispense Iv "gDNA - MM" (Col. 1, Row 4)
12	Dispense	1-8	2 µl Water free dispense New " RNA Norm" (Col. 1, Rows 1-8) , 1 option
13	Drop DiTis	1-8	Washstation 2Grid DiTi Waste
14	End Loop		"gDNA_Eraser"
15	User Prompt		"Incubate to 42°C for 2 min in PCR machine" sound : no
16	Begin Loop		3 times "Master Mix"
17	Get DiTis	1-8	DiTi 50ul LiHa
18	Aspirate	1	5.8 µl Water free dispense Iv "gDNA - MM" (Col. 2, Row 4)
19	Aspirate	2	5.8 µl Water free dispense Iv "gDNA - MM" (Col. 2, Row 4)
20	Aspirate	3	5.8 µl Water free dispense Iv "gDNA - MM" (Col. 2, Row 4)
21	Aspirate	4	5.8 µl Water free dispense Iv "gDNA - MM" (Col. 2, Row 4)
22	Aspirate	5	5.8 µl Water free dispense Iv "gDNA - MM" (Col. 2, Row 4)
23	Aspirate	6	5.8 µl Water free dispense Iv "gDNA - MM" (Col. 2, Row 4)
24	Aspirate	7	5.8 µl Water free dispense Iv "gDNA - MM" (Col. 2, Row 4)

25	Aspirate	8	5.8 µl Water free dispense Iv<br>"gDNA - MM" (Col. 2, Row 4)
26	Dispense	1-8	5.8 µl Water free dispense New<br>" RNA Norm" (Col. 1, Rows 1-8) , 1 option
27	Drop DiTis	1-8	Washstation 2Grid DiTi Waste
28	End Loop		"Master Mix"
29	User Prompt		"Incubate plate to 42°C for 15New -> 3 min to 95°C in PCR machine "<br>sound : no

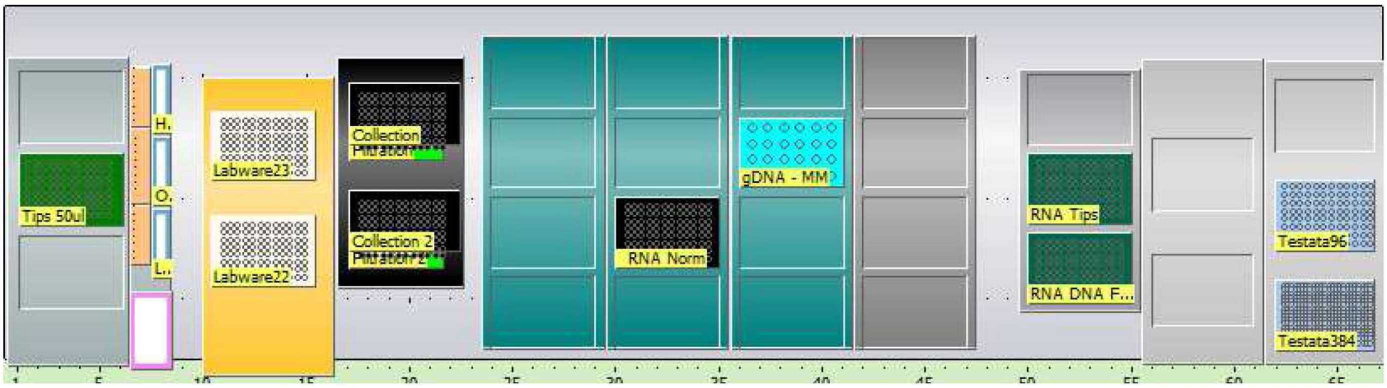

Supplement: Supplementary file 3 — Supplementary Material 3: Supplementary Material 3. Automated workflow cDNA synthesis script [file 12896_2024_831_MOESM3_ESM.pdf]
