# Supplementary material for: Reaping the benefits of liquid handlers for high-throughput gene expression profiling in a marine model invertebrate
Source: BMC Biotechnol. 2024 Jan 19;24:4. doi: 10.1186/s12896-024-00831-y (PMC10799371; doi:10.1186/s12896-024-00831-y)

**Supplementary Material 4.** Automated workflow RT-qPCR 384-well plate loading.

Script : 384 qPCR Assembling
User : Giovanni Annona



| | | | |
|---|---|---|---|
| 1 | Wash Tips | 1-8 | 25 + 30 ml |
| 2 | User Prompt | | "Spin cDNA Plate"<br>sound : no |
| 3 | Group | | Plate 1 |
| 4 | Set DiTi position | | DiTi 50ul LiHa<br>Grid : 1, Site : 1, First position in labware : 1 |
| 5 | Begin Loop | | 8 times "Primers" |
| 6 | Get DiTis | 1-8 | DiTi 50ul LiHa |
| 7 | Aspirate | 1 | 14 µl >> Water free dispense Iv <<<br>"Mix-Primers" (Col. 1, Row 1) , 1 option |
| 8 | Aspirate | 2 | 14 µl >> Water free dispense Iv <<<br>"Mix-Primers" (Col. 1, Row 1) , 1 option |
| 9 | Aspirate | 3 | 14 µl >> Water free dispense Iv <<<br>"Mix-Primers" (Col. 1, Row 1) , 1 option |
| 10 | Aspirate | 4 | 14 µl >> Water free dispense Iv <<<br>"Mix-Primers" (Col. 1, Row 1) , 1 option |
| 11 | Aspirate | 5 | 14 µl >> Water free dispense Iv <<<br>"Mix-Primers" (Col. 1, Row 1) , 1 option |
| 12 | Aspirate | 6 | 14 µl >> Water free dispense Iv <<<br>"Mix-Primers" (Col. 1, Row 1) , 1 option |
| 13 | Aspirate | 7 | 14 µl >> Water free dispense Iv <<<br>"Mix-Primers" (Col. 1, Row 1) , 1 option |
| 14 | Aspirate | 8 | 14 µl >> Water free dispense Iv <<<br>"Mix-Primers" (Col. 1, Row 1) , 1 option |
| 15 | Dispense | 1-8 | 14 µl Water free dispense Iv<br>"qPCR Mix Plate1" (Col. 1, Rows 1-8) , 1 option |
| 16 | Drop DiTis | 1-8 | Washstation 2Grid DiTi Waste |
| 17 | End Loop | | "Primers" |
| 18 | Set DiTi position | | DiTi 50ul Filter LiHa<br>Grid : 1, Site : 2, First position in labware : 1 |
| 19 | Begin Loop | | 8 times "SYBR" |
| 20 | Get DiTis | 1-8 | DiTi 50ul Filter LiHa |
| 21 | Aspirate | 1 | 17 µl >> Water free dispense Iv <<<br>"Mix-Primers" (Col. 3, Row 1) |
| 22 | Aspirate | 2 | 17 µl >> Water free dispense Iv <<<br>"Mix-Primers" (Col. 3, Row 1) |
| 23 | Aspirate | 3 | 17 µl >> Water free dispense Iv <<<br>"Mix-Primers" (Col. 3, Row 1) |
| 24 | Aspirate | 4 | 17 µl >> Water free dispense Iv <<<br>"Mix-Primers" (Col. 3, Row 1) |

| Step | Command | Tips | Parameters |
|---|---|---|---|
| 25 | Aspirate | 5 | 17 µl >> Water free dispense Iv << "Mix-Primers" (Col. 3, Row 1) |
| 26 | Aspirate | 6 | 17 µl >> Water free dispense Iv << "Mix-Primers" (Col. 3, Row 1) |
| 27 | Aspirate | 7 | 17 µl >> Water free dispense Iv << "Mix-Primers" (Col. 3, Row 1) |
| 28 | Aspirate | 8 | 17 µl >> Water free dispense Iv << "Mix-Primers" (Col. 3, Row 1) |
| 29 | Dispense | 1-8 | 17 µl Water free dispense Iv "qPCR Mix Plate1" (Col. 1, Rows 1-8) , 1 option |
| 30 | Drop DiTis | 1-8 | Washstation 2Grid DiTi Waste |
| 31 | End Loop | | "SYBR" |
| 32 | Begin Loop | | 7 times "cDNA" |
| 33 | Get DiTis | 1-8 | DiTi 50ul LiHa |
| 34 | Aspirate | 1 | 3.5 µl Water free dispense Iv "cDNA" (Col. 1, Row 1) , 1 option |
| 35 | Aspirate | 2 | 3.5 µl >> Water free dispense Iv << "cDNA" (Col. 1, Row 1) , 1 option |
| 36 | Aspirate | 3 | 3.5 µl >> Water free dispense Iv << "cDNA" (Col. 1, Row 1) , 1 option |
| 37 | Aspirate | 4 | 3.5 µl >> Water free dispense Iv << "cDNA" (Col. 1, Row 1) , 1 option |
| 38 | Aspirate | 5 | 3.5 µl >> Water free dispense Iv << "cDNA" (Col. 1, Row 1) , 1 option |
| 39 | Aspirate | 6 | 3.5 µl >> Water free dispense Iv << "cDNA" (Col. 1, Row 1) , 1 option |
| 40 | Aspirate | 7 | 3.5 µl >> Water free dispense Iv << "cDNA" (Col. 1, Row 1) , 1 option |
| 41 | Aspirate | 8 | 3.5 µl >> Water free dispense Iv << "cDNA" (Col. 1, Row 1) , 1 option |
| 42 | Dispense | 1 | 3.5 µl >> Water free dispense Iv << "qPCR Mix Plate1" (Col. 1, Row 1) , 1 option |
| 43 | Dispense | 2 | 3.5 µl >> Water free dispense Iv << "qPCR Mix Plate1" (Col. 2, Row 1) , 1 option |
| 44 | Dispense | 3 | 3.5 µl >> Water free dispense Iv << "qPCR Mix Plate1" (Col. 3, Row 1) , 1 option |
| 45 | Dispense | 4 | 3.5 µl >> Water free dispense Iv << "qPCR Mix Plate1" (Col. 4, Row 1) , 1 option |
| 46 | Dispense | 5 | 3.5 µl >> Water free dispense Iv << "qPCR Mix Plate1" (Col. 5, Row 1) , 1 option |
| 47 | Dispense | 6 | 3.5 µl >> Water free dispense Iv << "qPCR Mix Plate1" (Col. 6, Row 1) , 1 option |
| 48 | Dispense | 7 | 3.5 µl >> Water free dispense Iv << "qPCR Mix Plate1" (Col. 7, Row 1) , 1 option |

| | | | |
|---|---|---|---|
| 49 | Dispense | 8 | 3.5 µl >> Water free dispense lv << "qPCR Mix Plate1" (Col. 8, Row 1) , 1 option |
| 50 | Drop DiTis | 1-8 | Washstation 2Grid DiTi Waste |
| 51 | End Loop | "cDNA" | |
| 52 | Group | H2O NC | |
| 53 | Get DiTis | 1-8 | DiTi 50ul LiHa |
| 54 | Aspirate | 1 | 3.5 µl >> Water free dispense lv << "Mix-Primers" (Col. 3, Row 4) |
| 55 | Aspirate | 2 | 3.5 µl >> Water free dispense lv << "Mix-Primers" (Col. 3, Row 4) |
| 56 | Aspirate | 3 | 3.5 µl >> Water free dispense lv << "Mix-Primers" (Col. 3, Row 4) |
| 57 | Aspirate | 4 | 3.5 µl >> Water free dispense lv << "Mix-Primers" (Col. 3, Row 4) |
| 58 | Aspirate | 5 | 3.5 µl >> Water free dispense lv << "Mix-Primers" (Col. 3, Row 4) |
| 59 | Aspirate | 6 | 3.5 µl >> Water free dispense lv << "Mix-Primers" (Col. 3, Row 4) |
| 60 | Aspirate | 7 | 3.5 µl >> Water free dispense lv << "Mix-Primers" (Col. 3, Row 4) |
| 61 | Aspirate | 8 | 3.5 µl >> Water free dispense lv << "Mix-Primers" (Col. 3, Row 4) |
| 62 | Dispense | 1 | 3.5 µl >> Water free dispense lv << "qPCR Mix Plate1" (Col. 1, Row 8) |
| 63 | Dispense | 2 | 3.5 µl >> Water free dispense lv << "qPCR Mix Plate1" (Col. 2, Row 8) |
| 64 | Dispense | 3 | 3.5 µl >> Water free dispense lv << "qPCR Mix Plate1" (Col. 3, Row 8) |
| 65 | Dispense | 4 | 3.5 µl >> Water free dispense lv << "qPCR Mix Plate1" (Col. 4, Row 8) |
| 66 | Dispense | 5 | 3.5 µl >> Water free dispense lv << "qPCR Mix Plate1" (Col. 5, Row 8) |
| 67 | Dispense | 6 | 3.5 µl >> Water free dispense lv << "qPCR Mix Plate1" (Col. 6, Row 8) |
| 68 | Dispense | 7 | 3.5 µl >> Water free dispense lv << "qPCR Mix Plate1" (Col. 7, Row 8) |
| 69 | Dispense | 8 | 3.5 µl >> Water free dispense lv << "qPCR Mix Plate1" (Col. 8, Row 8) |
| 70 | Drop DiTis | 1-8 | Washstation 2Grid DiTi Waste |
| 71 | Group End | H2O NC | |
| 72 | Group End | Plate 1 | |

| | | |
|---|---|---|
| 73 | User Prompt | "Spin Mix Plates"<br>sound : no |
| 74 | Wizard | ReplicateWizard (...) |
| 75 | Set Variable | iterationCount = 0 |
| 76 | Begin Loop | 8 times "LoopSourceCols" |
| 77 | Begin Loop | 3 times "LoopReplications" |
| 78 | Condition | iterationCount % 3 <> 0<br>SkipLabel0_6336B5BD |
| 79 | Get DiTis | 1-8 DiTi 50ul LiHa |
| 80 | Comment | SkipLabel0_6336B5BD |
| 81 | Aspirate | 1-8 10 µl >> Water free dispense New <<<br>"qPCR Mix Plate1" (Col. 1, Rows 1-8) , 1 option |
| 82 | Dispense | 1-8 10 µl Water dry contact High density plate<br>"384 Plate" (Col. 1, Rows 1-8) , 2 options |
| 83 | Condition | (iterationCount + 1) % 3 <> 0<br>SkipLabel1_6336B5BD |
| 84 | Drop DiTis | 1-8 Washstation 2Grid DiTi Waste |
| 85 | Comment | SkipLabel1_6336B5BD |
| 86 | Set Variable | iterationCount = iterationCount + 1 |
| 87 | End Loop | "LoopReplications" |
| 88 | End Loop | "LoopSourceCols" |
| 89 | Wizard End | |

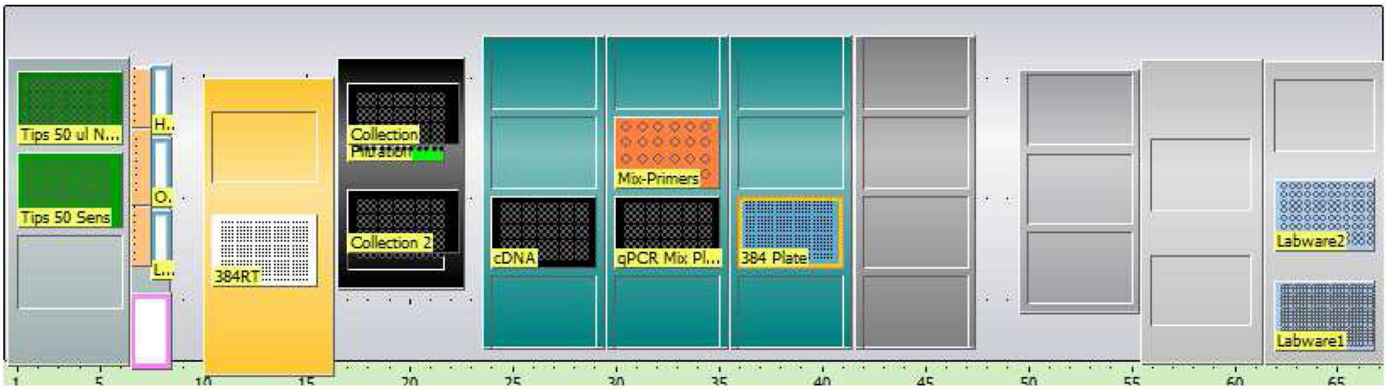

Supplement: Supplementary file 4 — Supplementary Material 4: Supplementary Material 4. Automated workflow RT-qPCR 384-well plate loading [file 12896_2024_831_MOESM4_ESM.pdf]
