# Supplementary material for: Reaping the benefits of liquid handlers for high-throughput gene expression profiling in a marine model invertebrate
Source: BMC Biotechnol. 2024 Jan 19;24:4. doi: 10.1186/s12896-024-00831-y (PMC10799371; doi:10.1186/s12896-024-00831-y)

Supplementary Material 5. Full-length gel electrophoresis reported in Figure 3 “Quality control of RNA extraction and cDNA synthesis using manual and automated procedures”. (A and B) Full-length of the RNA cropped gel reported in Panel A and Panel D of the Figure 3, respectively. (C and D) Full-length of the PCR cropped gel reported in Panel B and in Panel E of Figure 3, respectively, that **correspond to the lower part** of the full-length gels.

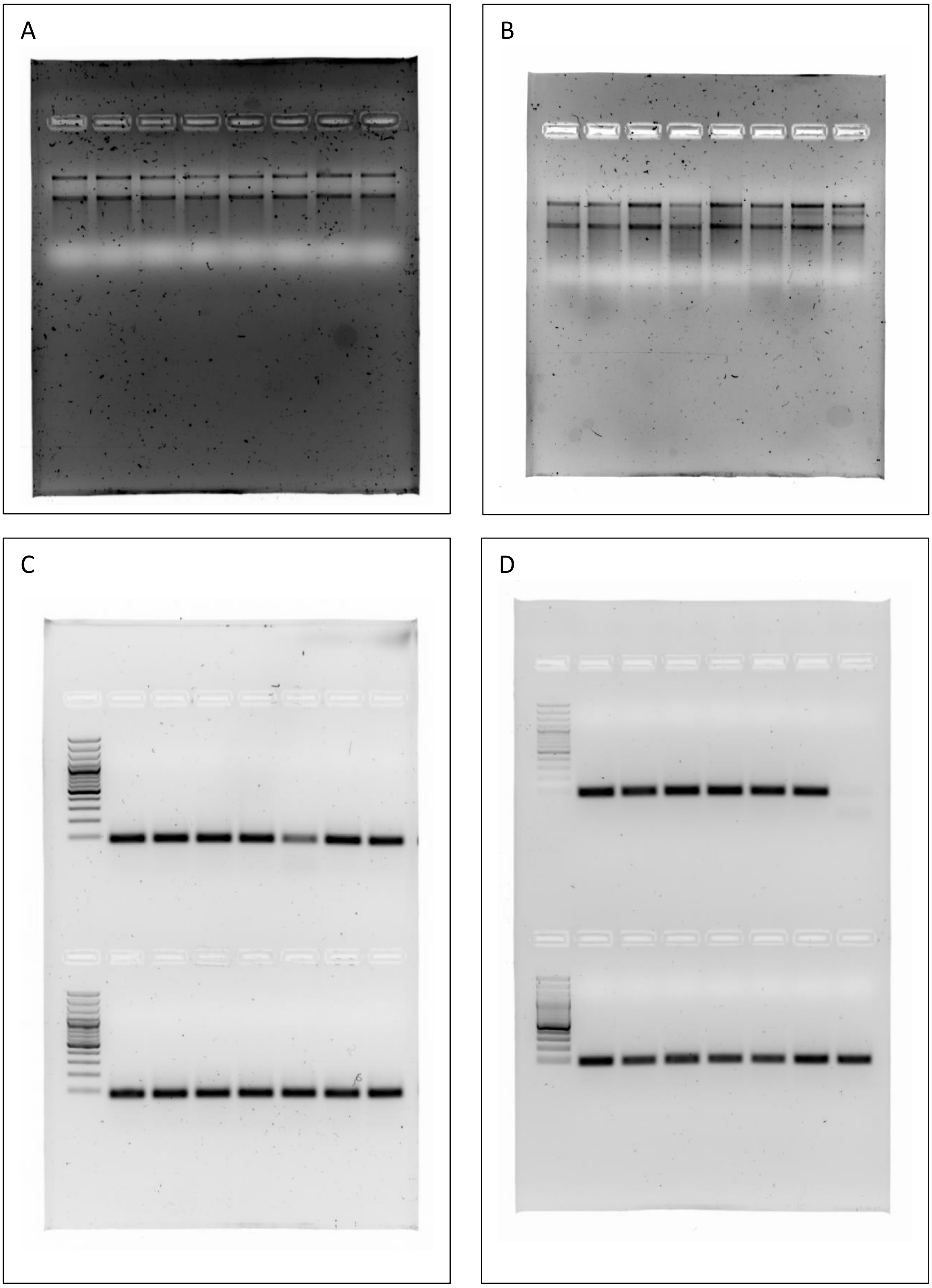

Supplement: Supplementary file 5 — Supplementary Material 5: Supplementary Material 5. Full-length gel electrophoresis reported in Figure 3 “Quality control of RNA extraction and cDNA synthesis using manual and automated procedures”. (A and B) Full-length of the RNA cropped gel reported in Panel A and Panel D of the Figure 3, respectively. (C and D) Full-length of the PCR cropped gel reported in Panel B and in Panel E of Figure 3, respectively, that correspond to the lower part of the full-length gels [file 12896_2024_831_MOESM5_ESM.pdf]
